# Remodeling of Aorta Extracellular Matrix as a Result of Transient High Oxygen Exposure in Newborn Rats: Implication for Arterial Rigidity and Hypertension Risk

**DOI:** 10.1371/journal.pone.0092287

**Published:** 2014-04-17

**Authors:** Fanny Huyard, Catherine Yzydorczyk, Michele M. Castro, Anik Cloutier, Mariane Bertagnolli, Hervé Sartelet, Nathalie Germain, Blandine Comte, Richard Schulz, Denis DeBlois, Anne Monique Nuyt

**Affiliations:** 1 Sainte-Justine University Hospital Research Center, Department of Paediatrics, Université de Montréal, Montreal, Québec, Canada; 2 Departments of Pediatrics & Pharmacology, Cardiovascular Research Centre, University of Alberta, Edmonton, Alberta, Canada; 3 Sainte-Justine University Hospital Research Center, Department of Pathology, Université de Montréal, Montreal, Québec, Canada; 4 Unit of Human Nutrition UMR 1019, INRA, Research Centre of Clermont-Ferrand/Theix, Saint-Genès-Champanelle, France; 5 Faculty of Pharmacy, Université de Montréal, Montreal, Quebec, Canada; University of Giessen Lung Center, Germany

## Abstract

Neonatal high-oxygen exposure leads to elevated blood pressure, microvascular rarefaction, vascular dysfunction and arterial (aorta) rigidity in adult rats. Whether structural changes are present in the matrix of aorta wall is unknown. Considering that elastin synthesis peaks in late fetal life in humans, and early postnatal life in rodents, we postulated that transient neonatal high-oxygen exposure can trigger premature vascular remodelling. Sprague Dawley rat pups were exposed from days 3 to 10 after birth to 80% oxygen (vs. room air control) and were studied at 4 weeks. Blood pressure and vasomotor response of the aorta to angiotensin II and to the acetylcholine analogue carbachol were not different between groups. Vascular superoxide anion production was similar between groups. There was no difference between groups in aortic cross sectional area, smooth muscle cell number or media/lumen ratio. In oxygen-exposed rats, aorta elastin/collagen content ratio was significantly decreased, the expression of elastinolytic cathepsin S was increased whereas collagenolytic cathepsin K was decreased. By immunofluorescence we observed an increase in MMP-2 and TIMP-1 staining in aortas of oxygen-exposed rats whereas TIMP-2 staining was reduced, indicating a shift in the balance towards degradation of the extra-cellular matrix and increased deposition of collagen. There was no significant difference in MMP-2 activity between groups as determined by gelatin zymography. Overall, these findings indicate that transient neonatal high oxygen exposure leads to vascular wall alterations (decreased elastin/collagen ratio and a shift in the balance towards increased deposition of collagen) which are associated with increased rigidity. Importantly, these changes are present prior to the elevation of blood pressure and vascular dysfunction in this model, and may therefore be contributory.

## Introduction

Conditions during early life (pre- and early postnatal) can significantly impact adult health and disease, particularly the cardiovascular system. Intrauterine growth restriction and preterm birth have been associated with elevated blood pressure and increased arterial stiffness in children and adults, but the pathophysiology underlying these observations is incompletely known [1], [2].

The proportion of elastin versus collagen is a major determinant of arterial stiffness [3], a well-established and independent marker of cardiovascular risk. Stiffening of large central arteries, particularly the aorta, occurs during physiological as well as pathological processes such as aging and hypertension [4]. Changes in elastic arteries properties are observed in borderline hypertension in humans and in animal models of chronic hypertension at a young age when blood pressure is minimally different from controls, suggesting that these alteration of vascular wall properties can precede the development of high blood pressure [5], [6].

Elastin synthesis in the vessels peaks in late fetal life in humans [7] (and in the first postnatal days in rodents), decreases rapidly after birth and is minimal in the adult aorta. Elastin has a very long half-life and a slow turnover [8]. Elastin content of the aortic wall and other large conduit arteries is therefore determined relatively early during development, with a low capacity for synthesis thereafter; modification due to adverse conditions during the perinatal period could therefore have long lasting consequences. Collagen synthesis increases during intrauterine life and persists after birth; the proportion of collagen in vessels therefore increases with age and synthesis can be accelerated in pathological conditions such as hypertension [9].

In conduit vessels, vascular smooth muscle cells are surrounded by the highly structured extracellular matrix consisting largely of collagens types I and III, elastin, and proteoglycans [10]. Matrix metalloproteinases (MMPs) and tissue inhibitors of metalloproteinases (TIMPs) play a crucial role in the vascular wall changes associated with aging or conditions such as hypertension, by regulating extracellular matrix turnover and collagen metabolism [4];[11]–[15]. Along with MMPs, the cysteine proteases, cathepsins S and K, can contribute to the degradation of extracellular matrix proteins such as elastin and collagen, respectively [16].

Among the many factors implicated in adverse perinatal conditions and later life cardiovascular consequences, oxidative stress is an important common denominator. Preterm infants, who represent 8% of newborns, have decreased antioxidant defenses compared to babies born at term [17] and are exposed to high levels of oxygen (O_2_) both in intensive care and as compared to the intrauterine environment [17], [18].

We have previously shown that O_2_ exposure of newborn rats, a well-established model of prematurity-related O_2_ injury, leads in early adulthood to elevated blood pressure (present by 7–8 weeks of life), endothelial dysfunction with enhanced superoxide production, and increased pulse wave velocity (indicative of arterial stiffness) [19]–[21]. We hypothesized that transient O_2_ exposure in neonatal period can alter aortic wall composition and remodeling, independently of increased blood pressure, vascular dysfunction and oxidative stress with the postulate that an imbalance in favor of vascular stiffening elements would prevail and be present early in life.

## Materials and Methods

### Animals

Animals were used according to a protocol approved by the Animal Care Committee of the CHU Sainte-Justine in accordance with the principles of the Guide for the Care and Use of Experimental Animals of the Canadian Council on Animal Care. Briefly, Sprague-Dawley rat pups (Charles River, St.-Constant, Québec, Canada) were maintained in 80% O_2_ (O_2_-exposed; by a mixture of medical grade 100% O_2_ and room air measured with an oxycycler A82OCV, Biospherix) or in room air (control) from postnatal day 3 to 10 of life, as reported [19]. Male O_2_-exposed and control rats were studied at 4 weeks.

### Experimental procedures

At 4 weeks, blood pressure measurements were obtained; rats were sacrificed after anaesthesia with intraperitoneal ketamine (Ayerst, Montreal, QC, Canada; 65 mg/kg) and xylazine (Bayer, Montreal, QC, Canada; 7 mg/kg) and thoracic aorta was sampled. All experiments were realized in *n* = 6 animals per group, from 3 litters per group (i.e. 2 animals studied per litter).

### Blood pressure measurement

After habituation for 1 week to the equipment and handler, systolic blood pressure (SBP) was assessed by tail-cuff plethysmography (50-001 Rat Tail Blood Pressure System, Harvard Apparatus, Holliston, MA).

### *Ex vivo* vascular reactivity studies

Freshly excised aortic rings in O_2_-exposed and control groups were studied as described [22]. Briefly, aortas were placed in ice cold modified Krebs bicarbonate solution of the following composition (in mM): 118 NaCl, 4.7 KCl, 25 NaHCO_3_, 2.5 CaCl_2_, 1.2 MgSO_4_, 1.2 KH_2_PO_4_, and 11 dextrose. Aortas were cleaned of fat and cut precisely into rings of equal length (4 mm). Four to eight rings from each rat were used per experiment and results were averaged. Rings were suspended horizontally between two stainless-steel wires in organ chambers that contained 20 ml of Krebs maintained at 37°C and aerated continuously with 95% O_2_ and 5% CO_2_. The tension of the preparations was recorded with a linear force transducer on a computerized data acquisition system (Kent Scientific, Litchfield, CT). The rings were progressively stretched to a preload tension of 19.0 mN and allowed to equilibrate for 30 min with frequent washing and tension adjustments. After stabilization, rings were repeatedly exposed to KCl (80 mM) to test their viability and to determine a standard contractile response for each ring. Cumulative concentration-response curves were then generated with the addition of 1 pM to 1 µM angiotensin II (Sigma Chemical, St Louis, MO) to induce vasoconstriction. For determining endothelium-dependent vasodilatory response to carbachol (Sigma Chemical, St Louis, MO; 100 nM to 100 µM), rings were contracted with U46619 (thromboxane A2 mimetic, Sigma-Aldrich, St Louis, MO; 0.3 µM; 15 minutes prior to adding carbachol).

### Vascular (aorta) superoxide production

Aortic superoxide levels were evaluated in O2-exposed and control groups using the oxidative fluorescent dye hydroethidine (2 µM) as reported [19]. Briefly, unfixed frozen aorta segments were cut into 12-µm-thick sections with a cryostat (Microm Cryostat, Waldorf, Germany) at −20°C and thaw-mounted on microscope slides (Superfrost, VWR Scientific, Pittsburgh, PA). Hydroethidine (2 µM) was applied to each tissue section and coverslipped. Slides were then incubated in a light-protected humidified chamber at 37°C for 30 min. Images were obtained with a laser scanning confocal microscope (LSM 510 laser scanning microscope; Zeiss) equipped with an argon laser. Fluorescence was detected with a 514-nm longpass filter. Digital images were collected. O_2_-exposed and control rat samples were treated in parallel for each condition and collected using identical conditions (exposure time, gain, and light intensity). Fluorescence was evaluated with the ImageJ software (http://rsbweb.nih.gov/ij) from at least 4 aortic sections per animal.

### Thoracic aorta wall composition and structure

In current study we aimed to examine vascular alterations in the thoracic aorta which is considered an elastic artery, with more elastic fibers, lower collagen content and is more compliant than the abdominal aorta [23]. Further, in a rodent model of chronic hypertension (spontaneously hypertensive rat) vascular wall changes were present in the thoracic aorta prior to blood pressure increase [6].

#### Elastin and collagen

Thoracic aortas were dissected, fixed in 10% neutral buffered formalin and paraffin-embedded. Sequential 5 µm sections were stained with Verhoeff-Van Gieson or Masson's trichrome stains for elastin and collagen fibres, respectively. On the histological sections, the area of the tunica media was calculated using image analysis software (Microscope Leiss Imager M1, Zeiss Germany. Axiovision 4.6, Zeiss Germany): a first area was delimited by the internal elastic membrane which forms a boundary between tunica intima and tunica media, and a second area was delimited by the boundary between tunica media and adventitia. The area of the tunica media was calculated by subtracting the first area from the second area. Secondly, the density of elastin fibres (Verhoeff-Van Gieson stain) and of collagen fibres (Masson's trichrome stain) were quantified using the image analysis software (as above) and the ratio of their respective densities to the surface of the tunica media area was calculated [24]. Three to four series of sections per rat were studied and averaged.

#### Western blotting

Aorta segments were homogenized in RIPA buffer containing proteases and phosphatases inhibitors. Antibodies against collagen I and collagen III, SMAD 3, TGF beta 1 (1/1000 dilution), cathpesin S (1/2000 dilution, all Abcam, Cambridge, MA, United States) and AT1 (1/1000 dilution, Santa Cruz Biotechnology, Santa Cruz, CA, United States) were used in this study. Antibody against β-actin (1/20 000) was used as control. Protein bands were developed with an enhanced chemiluminescence substrate (PerkinElmer Inc, Waltham, MA) and quantified using ImageJ 1.36b (http://rsbweb.nih.gov/ij/).

#### Cross sectional area and smooth muscle cell number

Fixed aortas were embedded in paraffin blocks. Aortic cross-sectional area and smooth muscle cell (SMC) number were evaluated in 3-µm sections of aorta, stained with hematoxylin and eosin. The tridimensional dissector method was used to evaluate the number of SMCs per unit of vessel length, using the adapted method of Mulvany et al [25] as described previously by deBlois et al [26]. Three consecutive sections were photographed at 400× magnification and analyzed as described [26]. Digital images were analyzed using National Institutes of Health Image J software 1.32 (public domain software, available at: http://rsb.info.nih.gov/nih-image/index.html).

### Immunofluorescence

Frozen sections of aorta (8 µm thick) were immunostained with: cathepsin S (goat anti-cathepsin S 1∶100, ab18822, Abcam Ltd, Cambridge, UK), cathepsin K (rabbit anti-cathepsin K 1∶100, ab19027, Abcam Ltd, Cambridge, UK), MMP-2 (rabbit anti-MMP-2 1∶50, ab37150, Abcam Ltd, Cambridge, UK), TIMP-1 (rabbit anti-TIMP-1∶100, ab770, Millipore, Billerica, MA, USA) or TIMP-2 (mouse anti-TIMP2 1∶100, ab1828, Abcam Ltd, Cambridge, UK), overnight at 4°C. The sections were then washed with PBS and incubated 1 h with Alexa Fluor-488 (donkey anti-goat IgG 1∶200 for cathepsin S; goat anti-rabbit IgG 1∶200 for cathepsin K, MMP-2, MMP-9, and TIMP-1, goat anti-mouse IgG 1∶200 for TIMP-2). For each antibody, all sections from O_2_-exposed and control rats were processed in parallel (same conditions). In order to quantify pixel fluorescence intensity on pictures, digital images were collected on at least four aortic sections per animal under the same conditions and the average value from all sections from each animal was used for comparison between groups, as previously published [20]. Digital images were analyzed using the software described above.

### Measurement of MMP activity by gelatin zymography [27]

Frozen aortas (5 mm segments; snap frozen in liquid nitrogen upon sampling) were crushed by percussion using a stainless steel piston and cylinder that were cooled to liquid nitrogen temperature. The resulting powder was diluted 1∶4 w/v in 50 mM Tris-HCl (pH 7.4) buffer containing 3.1 mM sucrose, 1 mM dithiothreitol, 10 µg/mL leupeptin, 10 µg/mL soybean trypsin inhibitor, 2 µg/mL aprotinin and protease inhibitor cocktail (P8340, Sigma Chemical, St Louis, MO). This solution was then homogenized by hand on ice using a motorized pellet pestle (Kontes-Sigma Aldrich, St Louis, MO) for 2 minutes. The homogenate was centrifuged at 10,000 g for 5 minutes at 4°C and the supernatant was kept on ice for biochemical analysis. Aortic protein content was determined by the bicinchoninic acid method (Sigma Chemical, St Louis, MO) using bovine serum albumin as a standard.

Non-heated samples were diluted with water in order to load a constant amount of protein per lane (10 µg from aorta homogenate). These samples were then subjected to electrophoresis on 8% polyacrylamide gels co-polymerized with gelatin (2 mg/mL, type A from porcine skin, Sigma Chemical, St Louis, MO). After 1.5 h of electrophoresis, the gels were incubated for 1 h at room temperature in a 2.5% v/v Triton X-100 solution, and incubated at 37°C for 20 h in incubation buffer (50 mM Tris-HCl, 150 mM NaCl, 5 mM CaCl_2_ and 0.05% NaN_3_). The gels were stained with 0.05% Coomassie Brilliant Blue G-250 in a mixture of methanol∶acetic acid∶water (2.5∶1∶6.5 v/v), and then destained in aqueous 4% v/v methanol and 8% v/v acetic acid. Gelatinolytic activities were detected as unstained bands against the background of Coomassie blue-stained gelatin, assayed by densitometry using ImageJ 1.36b (National Institutes of Health, USA). Intergel analysis was possible after normalization of gelatinolytic activity with an internal standard (conditioned medium from phorbol ester activated HT-1080 cells, American Type Culture Collection).

### Statistical analysis

Values are expressed as the mean ± SEM. Ex vivo concentration-response curves to AngII and carbachol were analyzed by computer fitting to a four-parameter sigmoid curve using GraphPad Prism 5 software (GraphPad, San Diego, CA) to evaluate the EC50 and Emax (the maximum asymptote of the curve). Analyses of differences within and between groups were performed using Student's t test for unpaired observations. Statistical significance was accepted at p<0.05.

## Results

### Blood pressure, aortic vasomotor response and vascular superoxide production

**Figure 1:**
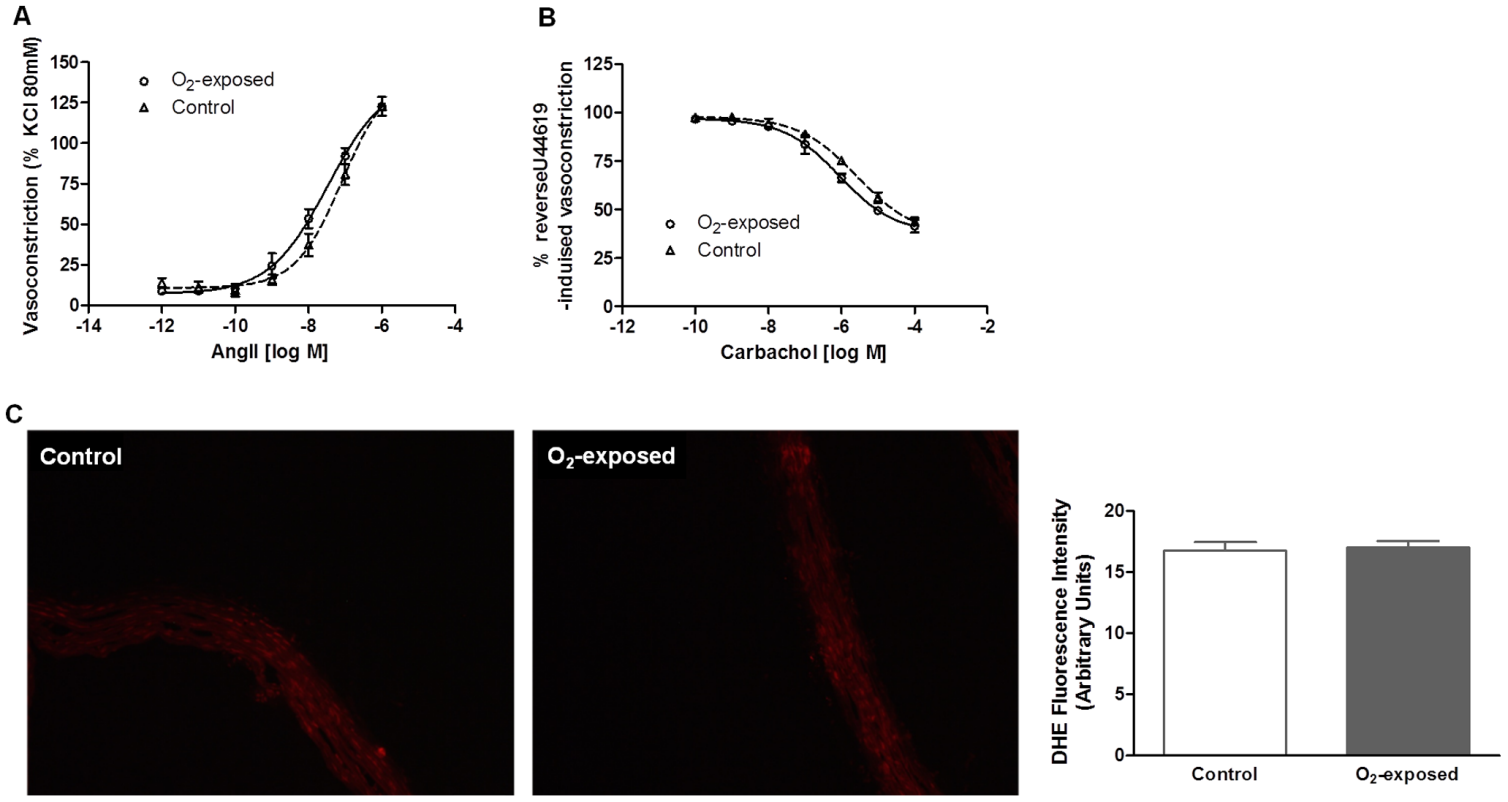
Impact of neonatal oxygen exposure on vascular superoxide production and vasomotricity in 4 weeks-old rats. Vasomotor responses of aortic rings from 4 weeks-old control versus O_2_-exposed rats to angiotensin II (constriction is expressed relative (percentage) to the response elicited by KCl (80 mM)) (**A**) and to acetylcholine analogue carbachol (vasodilatation is expressed as percent reversal of U46619 (0.3 µM)-induced vasoconstriction) (**B**). **C**: Superoxide anion production in aortas of O_2_-exposed rats. Representative sections (×20 magnification) after treatment of the aortas with hydroethidine (2 µM) in control versus O_2_-exposed rats and histogram of compiled data. Data are mean ± SEM of n = 5 rats per group.

These first experiments investigated whether transient neonatal oxygen exposure is associated with changes in aortic vasomotor response and vascular superoxide production at 4 weeks, prior to blood pressure elevation in this model [19]. Systolic blood pressure (132±2 mmHg O_2_-exposed vs. 133±2 mmHg control), diastolic blood pressure (100±8 mmHg O_2_-exposed vs. 91±12 mmHg control), mean blood pressure (111±6 mmHg O_2_-exposed vs. 105±9 mmHg control), angiotensin II-induced vasoconstriction (Fig. 1A), carbachol-induced vasodilatation (Figure 1B) and vascular superoxide production (Figure 1C) were not different between 4 weeks old O_2_-exposed and control groups (p>0.05).

### Aortic wall structure, and collagen and elastin content

**Figure 2:**
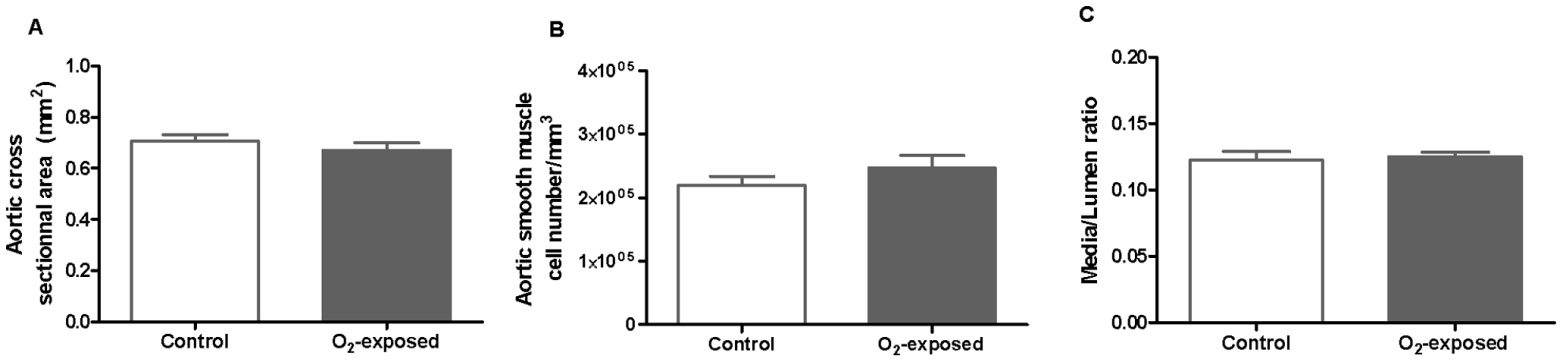
Neonatal oxygen exposure does not lead to hyperplasia or hypertrophy in aortic media of 4 weeks-old rats. Aortic cross-sectional area (**A**), aortic smooth muscle cell number (**B**) and Media/Lumen ratio (**C**) in 4 weeks-old control versus O_2_-exposed rats. Data are mean ± SEM of n = 6 rats per group.

To determine whether transient neonatal oxygen exposure induced aortic wall structure changes that could be present prior to blood pressure increase and vascular dysfunction present in adults [19], we assessed smooth muscle cells (i.e. aortic cross sectional area for hypertrophy and smooth muscle cells number for hyperplasia), and collagen and elastin content at 4 weeks of age. There was no difference in the aortic cross-sectional area, aortic smooth muscle cells number and media to lumen ratio between control and O_2_-exposed rats (Figure 2).

**Figure 3:**
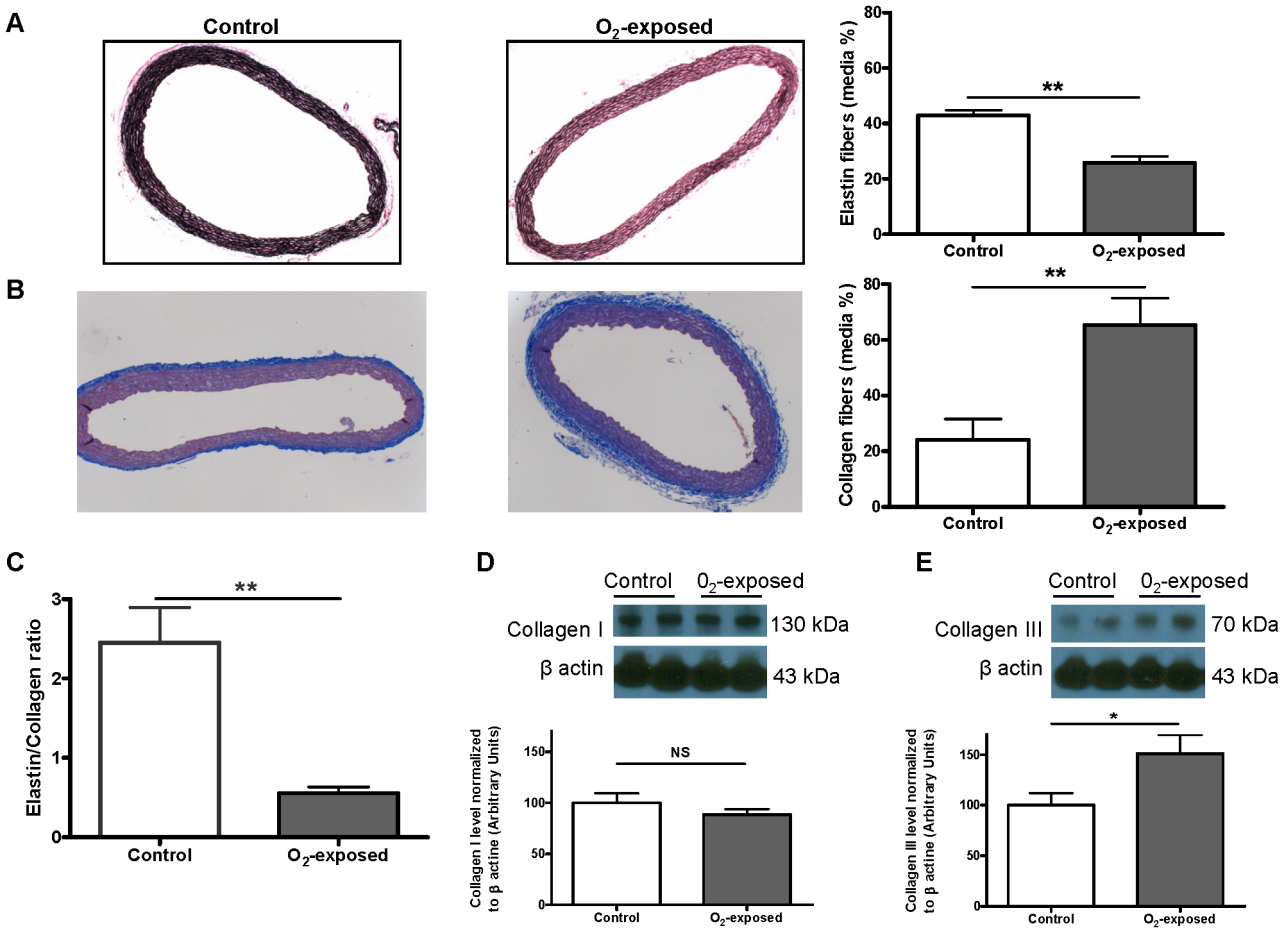
Neonatal oxygen exposure decreased elastin and increased collagen content in aortic media of 4 weeks-old rats. Representative photomicrographs (×10 magnification) of aorta sections from 4 weeks-old control versus O_2_-exposed rats stained with (**A**) Verhoeff-Van Gieson for elastin fibers and (**B**) Masson's Trichrome for collagen fibers with respective compiled density (expressed relative (percentage) to media area), and (**C**) the elastin/collagen ratio. Representative immunoblots and histogram of compiled data of (**D**) collagen I and (**E**) collagen III protein levels in aorta from 4 weeks-old control versus O_2_-exposed rats. Data are mean ± SEM of n = 6 rats per group. NS: non-significant, * p<0.05, ** p<0.01.

While elastin fiber density (Verhoeff staining) was significantly decreased in aortas of O_2_-exposed versus control rats, collagen fiber density (Masson's trichrome) was significantly increased, with a significant reduction in the elastin/collagen ratio in the O_2_-exposed group (Figure 3A–E). Protein levels of collagen III, but not collagen I, were significantly increased in O_2_-exposed rats (Figure 3F and 3G). No difference was observed between groups in the protein expression of vascular profibrogenic angiotensin II AT1 receptor, as well as in TGFβ and SMAD-3, downstream signalling cascade factors that can be triggered by inflammation/MMP-2 pathway.

### MMPs and cathepsins in aorta of O_2_-exposed rats by immunofluorescent staining

**Figure 4:**
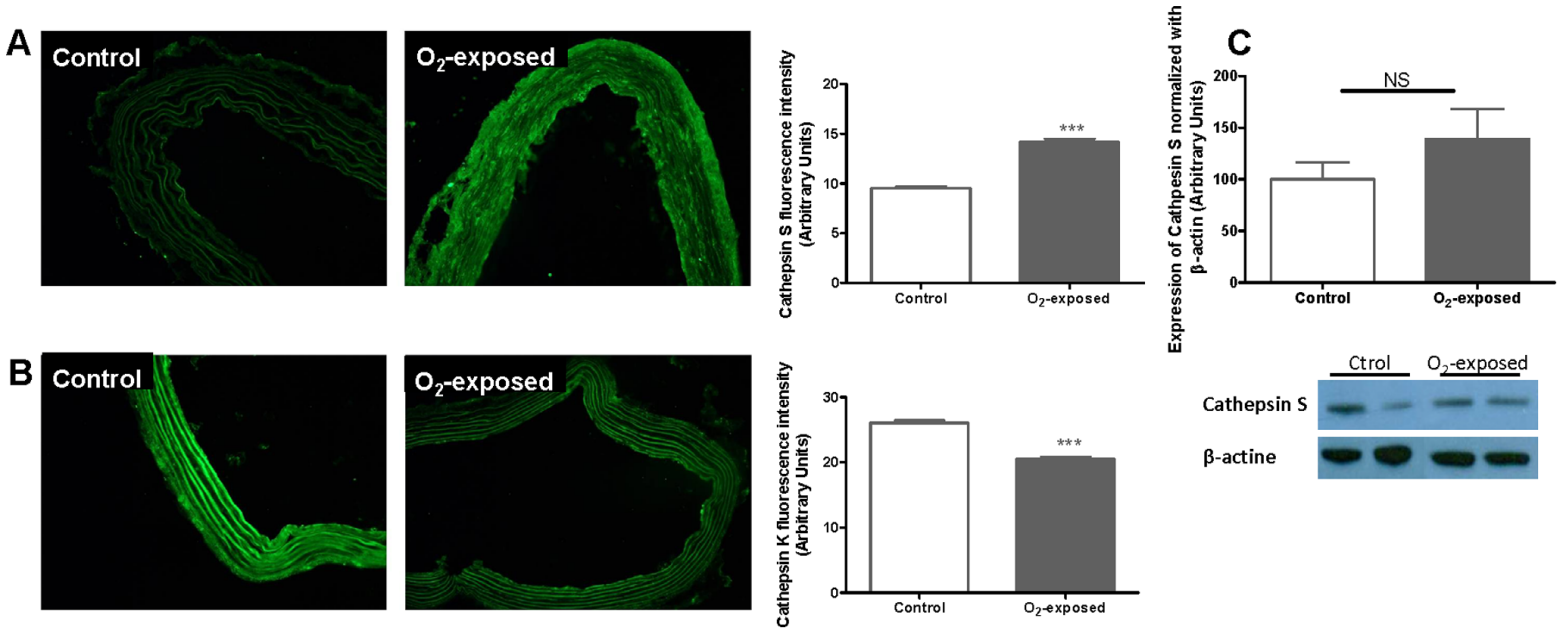
Neonatal oxygen exposure increased cathepsin S and decreased cathepsin K in aortic media of 4 weeks-old rats. Representative photomicrographs (×20 magnification) and histogram of compiled data of cathepsin S (**A**) and cathepsin K (**B**) immunofluorescence of aortic sections from 4 weeks old control versus O_2_-exposed rats. Representative immunoblot and histogram of compiled data of cathepsin S (**C**) protein expression in aorta from 4 weeks-old control versus O_2_-exposed rats. Data are mean ± SEM of n = 6 rats per group. *** p<0.001.

**Figure 5:**
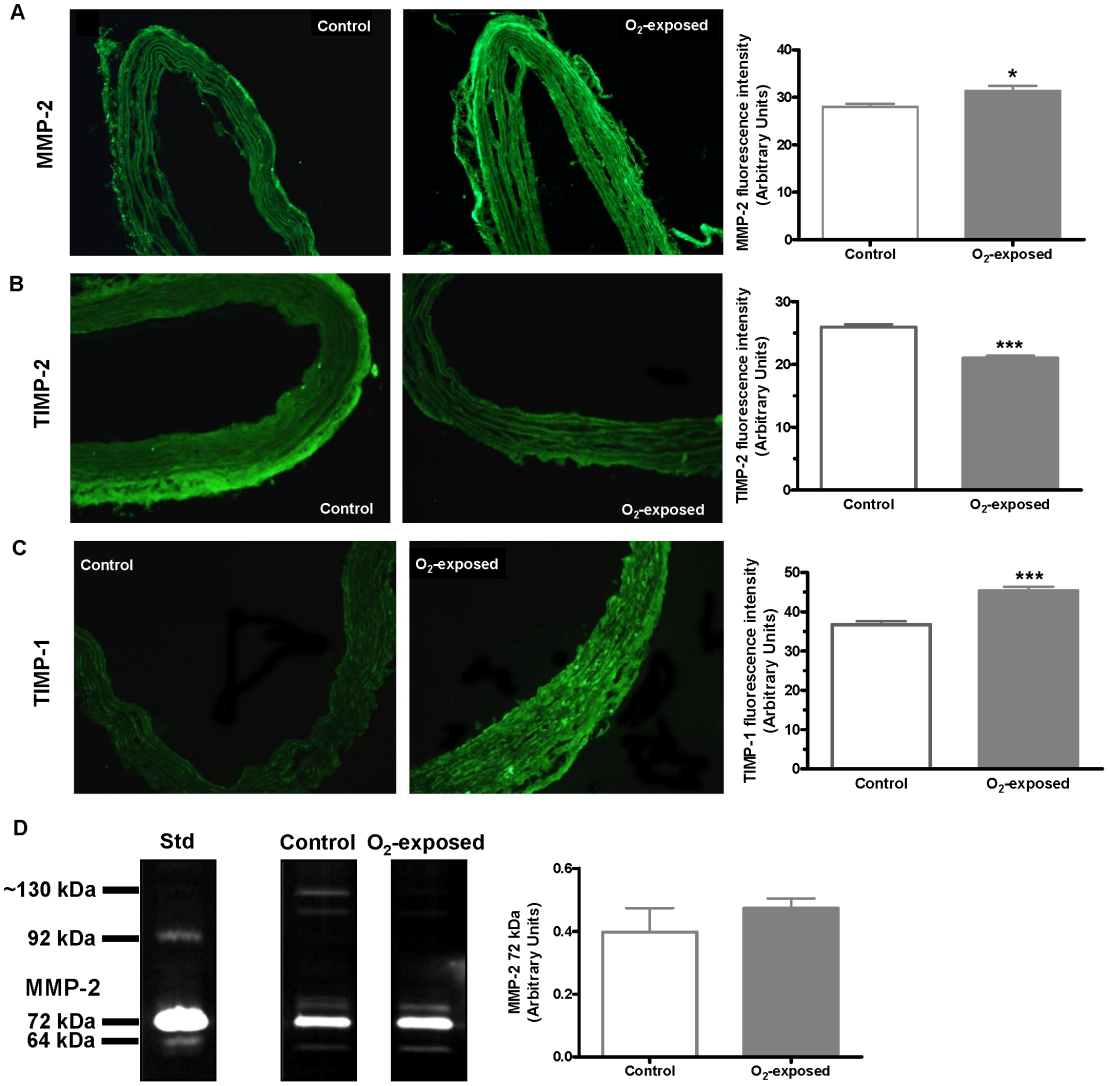
Impact of neonatal oxygen exposure on MMP-2, and TIMP-2 and TIMP-1 in aortic media of 4 weeks-old rats. Representative photomicrographs (×20 magnification) and histogram of compiled data of matrix metalloproteinase (MMP)-2 (**A**), tissue inhibitor of metalloproteinase (TIMP)-2 (**B**) and TIMP-1 (**C**) immunofluorescence of aortic sections from 4 weeks old control versus O_2_-exposed rats. (**D**) Representative gel and histogram of compiled data of 72 kDa MMP-2 activity by gelatin zymography analysis in aortas from 4 weeks old control versus O_2_-exposed rats. Data are mean ± SEM of n = 6 rats per group. * p<0.05, *** p<0.001.

In order to investigate the mechanisms potentially involved in the vascular changes observed, we assessed the enzymes Cathepsin S and K, and MMPs known to play a role in vascular wall structure remodeling. Immunohistofluorescence analysis showed increased levels of elastinolytic cathepsin S and reduced levels of collagenolytic cathepsin K in aortas from O_2_-exposed versus control animals (Figure 4A–B). Difference in Cathepsin S protein expression assessed by Western blot did not reach statistical significance (Figure 4C). Immunofluorescent staining for MMP-2 and TIMP-1 was significantly increased in aorta from O_2_-exposed versus control animals (Figure 5A and 5D). However using zymography, no difference was observed in the 72 kDa MMP-2 activity between the groups (Figure 5E). Immunofluorescence for TIMP-2 was significantly decreased in aorta from the O_2_-exposed rats compared to control group (Figure 5B and 5C). No bands corresponding to MMP-9 were detected in aorta samples from either group.

## Discussion

The current study shows that high oxygen exposure during the neonatal period alters aorta wall structure through the accumulation of collagen, decreased elastin and modifications of extracellular matrix components cathepsin S and cathepsin K, which can all contribute to extracellular matrix remodeling. The basal levels of MMPs and TIMPs were also modified favouring collagen increases. Importantly, these vascular wall changes are present at 4 weeks, which is younger than the age at which we reported blood pressure elevation and vascular dysfunction in association with transient neonatal oxygen exposure, and could contribute to their development. Of note, in situ superoxide levels were measured using the oxidative fluorescent dye hydroethidine, as described previously [19]. A recent review [28] discuss limitations of hydroethidine to assess superoxide anion production and suggests that 2-OH-E+ seems not to be formed during the reaction between HE and other oxidants (ONOO–, ^•^OH, H_2_O_2_), therefore, because of other oxidative reactions of HE, this review suggests that 2-OH-E+ formation is only a qualitative and not a quantitative indicator of superoxide anion production. This limitation does not change current results considering no difference between fluorescence intensity was noted between groups.

### Impact of neonatal oxygen exposure on elastin, collagen and vascular wall structure

It is well established that hypertension is associated with vascular oxidative stress and dysfunction [29], [30], as well as to vascular wall changes and enhanced stiffness; however whether oxidative stress and vascular stiffness are cause or result of high blood pressure remains debated [31], [32]. We have shown previously that neonatal oxygen exposure led, in early adult life (from 7–8 weeks of age), to elevated blood pressure associated with an increased vascular superoxide production, vascular dysfunction (increased vasoconstriction to angiotensin II and impaired endothelium-mediated vasodilatation) and systemic capillary rarefaction [19]. The current results suggest that changes in the aorta structure triggered by neonatal oxygen exposure precede and could be primary elements in the later rise in blood pressure and vascular dysfunction.

Large arteries can play an important role in the pathophysiology of hypertensive disease through propagation of the pulse wave further into the tissues thereby contributing to tissue damage and inflammation [33]. The mechanical properties of large blood vessels are derived from elastin and collagen fibres [34]–[36]. The major mechanical properties related to blood vessel function are tensile stiffness, elasticity and compressibility. Elastin stabilizes the arterial structure, inhibits smooth muscle cell proliferation, regulating their organization and migration [37]. Collagen provides the tensile stiffness for the resistance against rupture, elastin dictates the elastic properties and, combined with collagen, prevents irreversible deformation of the vessel against pulsatile blood flow; proteoglycans contribute to the compressibility [38], [39]. Elastinolysis and collagenolysis play crucial roles in arterial remodeling and diseases [40]. Proteolytic degradation of elastic fibers leads to loss of tissue elasticity, which is associated with increased arterial stiffness and is a risk factor to the development of cardiovascular diseases [33], [41]. As elastin synthesis in the vessels is important in the prenatal period and decreases after birth, disruption of elastin synthesis at the end of gestation or in the event of preterm birth may have long-term consequences [7] [42].

Our data show that neonatal oxygen exposure leads to an increase in collagen and decrease in elastin fibres in the aorta, which is consistent with increased arterial stiffness observed in O_2_-exposed adults [21]. In humans, studies report increased aortic stiffness in children aged 7–14 years who were born moderately or very preterm [43]–[45]. In studies in which tissues such as lung and osteoblasts were examined, exposure of newborn rats to high concentration of O_2_ increased type I collagen synthesis and decreased elastin deposition [46]–[49]. Matrix proteins, such as collagen and elastin, can influence vascular smooth muscle cell activities leading to phenotypic modulation, migration and proliferation [50], [51]. However, in the current study, examination of the aortic cross-sectional area and the media/lumen ratio did not reveal any vascular hypertrophy. Similarly, no difference in the number of aortic smooth muscle cells was found between the groups, which suggests that there is no hyperplasia at 4 weeks. Supporting these findings, data from adult models of vascular diseases show that reactive oxygen species lead to enhance collagen deposition and vascular matrix remodelling; interestingly for this current study which was realized in young rats (4 weeks), reactive oxygen species activate fibroblasts to secrete elements such as extracellular matrix proteins prior to proliferation and vascular wall hypertrophy [52]. Alternatively, increase in reactive oxygen species may be associated with a defect in elastin synthesis as reactive oxygen species can accelerate tropoelastin (elastin precursor) degradation [53]. Overall, in the current observations, the relative contribution of decreased elastin vs. increased collagen deposition on later vascular stiffness and elevation of blood pressure is unknown.

Data from other models of developmental programming of hypertension and vascular dysfunction also report early changes in the structure and composition of large arteries. A deficit in elastin content along with increased collagen deposition and vascular smooth muscle cell hypertrophy in the aorta were reported at 2 months rats (young adults) exposed to nutrient restriction in utero (global caloric restriction or low protein diet) [54]–[56]. Interestingly, we reported that neonatal oxidative stress plays a key role in programming elevated blood pressure and vascular dysfunction in the low protein diet model [57]. In another study, preadolescent normotensive lambs (9 weeks) which had been delivered prematurely (at 0.9 gestation) show remodeling of the aortic wall with increased thickness, increased elastin deposition, reduced smooth muscle content but unchanged collagen content [24]. Differences between these studies and the current report are not all elucidated but can be related to differing type of stressor (restricted diet vs. oxygen), timing of insult, and species studied.

### Neonatal oxygen exposure and cathepsins

Cathepsin S has an important role in extracellular matrix degradation and smooth muscle cells invasion thereby playing a major role in atherosclerosis [58] as well as in angiogenesis [59]–[61]. Cathepsin K is a member of the lysosomal cysteine and aspartic proteinase family, and possesses unique collagenolytic activity with the ability to cleave collagen at multiple sites, to depolymerize collagen fibers and cleave triple helixes [62], [63]. Our results show an increase in the level of cathepsin S and a decrease in the level of cathepsin K in aortas from O_2_-exposed versus control rats, suggesting that altered levels of cathepsins may play a role in the observed vascular wall changes. Reports from the literature support this postulate. In adults with chronic kidney diseases, increased serum levels of cathepsin S and MMP-2 are associated with aortic stiffening [64]. In newborn mice, cathepsin S deficiency prevents elastin degradation and profibrotic changes, and protects from O_2_-induced lung injury [65]. Cathepsin S is present as small foci on the smooth muscle cells plasma membrane but not in the cytosol [66], which may explain the differences in expression observed using immunofluorescence of aortic sections but not with immunoblot of whole vessel lysate. Conversely, cathepsin K deficiency aggravates lung injury in high O_2_-exposed newborn mice [67]. Cathepsin K expression levels are significantly reduced in the lungs of premature infants with bronchopulmonary dysplasia [68]; in contrast, cathepsin K is upregulated in fibrotic adult lungs, suggesting a protective role against excessive collagen deposition in adult chronically diseased lungs [69]. To our knowledge, expression of cathepsins in the systemic vasculature during development has not yet been reported, either in physiological or pathological conditions.

### Neonatal oxygen exposure and MMPs and TIMPs

MMPs and their related TIMPs can regulate vascular extracellular matrix remodeling in both physiological and pathological processes [70]. Among MMPs, MMP-2 and MMP-9 cleave gelatin, collagen and elastin and are recognized as important contributing elements in cardiac and vascular diseases (reviewed by [70], [71]). MMPs are also involved in angiogenesis during embryonic development [72] or pathologies such as tumors and retinal neovascularization [73] [74]. MMP-2 has recognized elastase activity [75] and so may have a role in fragmentation of the elastic laminae. MMP-9 is expressed constitutively in the heart whereas its presence in other tissue is mostly linked to a cytokine-inducible expression in leucocytes and inflammatory cells [71]. Endogenous TIMPs prevent excessive degradation of extracellular matrix; TIMP-2 and TIMP-1 are considered to preferentially (but not exclusively) inhibit MMP-2 and MMP-9, respectively [71] [76].

In the aorta of O_2_-exposed animals, we observed an increase in MMP-2 and TIMP-1, with a decrease in TIMP-2. These results support the postulate that vascular wall changes observed after neonatal O_2_-exposure can be related to imbalance in the expression of MMPs/TIMPs resulting in elastolytic/profibrotic conditions. Interestingly, MMP-2 and cathepsin S work in concert in vascular extracellular matrix degradation [66].

The current findings are overall in agreement and supported by reports in the literature. Increased circulating levels of MMP-2 as well as of MMP-2/TIMP-2 ratio were reported in small for gestational age children and MMP-2 was positively correlated with systolic blood pressure [77]. Experimentally, impact of neonatal high O_2_ exposure on MMPs has been mainly reported in the lungs. Adult rats exposed to 85% O_2_ presented increased levels of MMP-2 and MMP-9 activities in both bronchoalveolar lavage fluid and type II cells [78]. An increase in TIMP-1 expression was observed in lungs from rat pups exposed to a hyperoxic environment [79]. Hypertensive adults showed reduced expression of MMPs inducers along with evidence of suppressed degradation of collagen I, suggesting enhanced collagen deposition [80]. In experimental models of hypertension and vascular diseases, MMP-2 and -9 expressions and/or activities were modulated by proinflammatory and oxidative stress stimuli [81]–[83]. The absence of detection of MMP-9 in current study most probably relates to low or absent inflammation in the aorta at 4 weeks of age and/or to a level of expression below our detection limits. Mechanisms underlying changes in MMPs/TIMPs as well as cathepsins expression after transient neonatal high O_2_ exposure are not known; potential avenues comprise cellular changes in redox status and epigenetic modifications.

## Conclusion

We have shown that a neonatal transient hyperoxic exposure results in early modification of aorta architecture, independently of an elevation in blood pressure. At 4 weeks of age, neither vascular dysfunction, or aortic oxidative stress tissue, or hyperplasia/hypertrophy of smooth muscle cells was present. However, the elastin/collagen ratio as well as the expression of key extracellular matrix degradation enzymes such as the cathepsins, MMPs and TIMPs were modified and indicated profibrosis. We postulate that these early changes can significantly contribute to the onset of vascular dysfunction and elevated blood pressure which are present in early adulthood following neonatal exposure to hyperoxic stress.
